# Precision Agriculture: Computer Vision-Enabled Sugarcane Plant Counting in the Tillering Phase

**DOI:** 10.3390/jimaging10050102

**Published:** 2024-04-26

**Authors:** Muhammad Talha Ubaid, Sameena Javaid

**Affiliations:** 1 Faculty of Information Technology, University of Central Punjab, Lahore P.O. Box 54000, Pakistan; l1s19mscs0003@ucp.edu.pk; 2 Faculty of Computer Sciences, School of Engineering Sciences, Bahria University, Karachi P.O. Box 75290, Pakistan

**Keywords:** faster R-CNN, object detection, plant counting, sugarcane counting, sugarcane detection

## Abstract

The world’s most significant yield by production quantity is sugarcane. It is the primary source for sugar, ethanol, chipboards, paper, barrages, and confectionery. Many people are affiliated with sugarcane production and their products around the globe. The sugarcane industries make an agreement with farmers before the tillering phase of plants. Industries are keen on knowing the sugarcane field’s pre-harvest estimation for planning their production and purchases. The proposed research contribution is twofold: by publishing our newly developed dataset, we also present a methodology to estimate the number of sugarcane plants in the tillering phase. The dataset has been obtained from sugarcane fields in the fall season. In this work, a modified architecture of Faster R-CNN with feature extraction using VGG-16 with Inception-v3 modules and sigmoid threshold function has been proposed for the detection and classification of sugarcane plants. Significantly promising results with 82.10% accuracy have been obtained with the proposed architecture, showing the viability of the developed methodology.

## 1. Introduction

In many different nations, sugarcane is considered a significant crop. In developing countries, sugarcane is cultivated from 450 to 500 per acre mounds. Cultivation factors like preliminary culturing, soil preparation, planting time, accessibility of water for irrigation, choice of fertilizers, the plant’s health and security, and reaping time altogether influence its production [1]. Some farmers utilize management-oriented models (MOM) to limit nitrate leaking, which increases their production [2]. The agriculture sector is now utilizing artificial intelligence (AI) models to estimate seasonal crop yield. An AI-based model can anticipate forthcoming yield in advance, helping farmers and stakeholders make early decisions.

**Figure 1:**
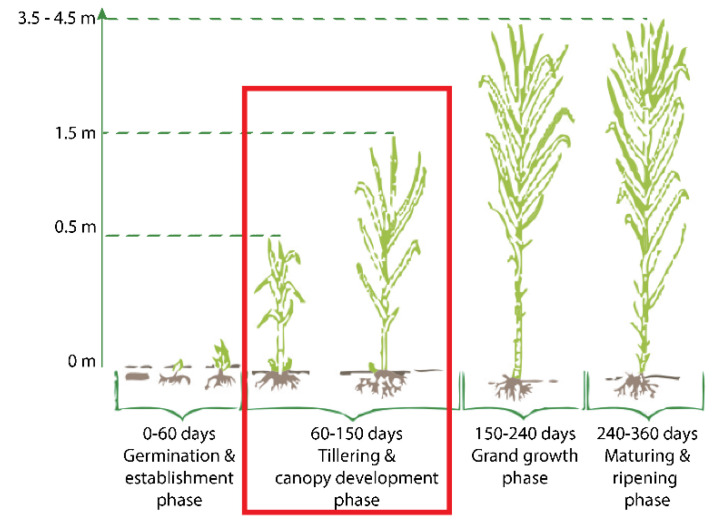
Phases of sugarcane plant [3].

There are four different growth phases that sugarcane goes through, as shown in Figure 1. The first phase is a germination and establishment phase. Internal and external factors influence the growth of the plants in this phase. External components like soil’s dampness, circulation of air, and the soil’s temperature, along with internal factors like sett supplement status, the bud’s health, and the moisture of sett decrease the sugar content. It begins from 7 to 10 days and usually goes on for 30 to 35 days. After 40 days, the second phase tillering begins and lasts up to 120 days. It is a repeated physiological cycle of underground branching from minimized nodal joints of the actual shoot. To fulfill the needs of a decent yield, tillering furnishes the product with a good stalk number. In a 12 month sugarcane process, grand growth, which is the third phase, starts from the 120th day and endures up to 270 days. Growth is considered a significant yield period, where the actual formation and extension of the cane happen. Under suitable conditions, the stalk develops quickly (about 4–5 internodes every month). Sugarcane enters its fourth stage of development in the last three months, between 270 to 360 days. It is called the ripening and outgrowing stage. In this stage, the synthesis and fast-growing of sugar take place, whereas vegetative development is diminished. In full-grown sugarcane, the bottom contains more parts of sugar than the top [3]. 

The sugarcane industries are keen on knowing the pre-harvest estimation of the sugarcane field. For this purpose, industries need a mechanism to make a prediction ahead of time on a particular piece of land where sugarcane is not fully grown to obtain an estimate that a specific land would produce this particular amount of sugarcane [4]. To date, farmers do not have any specific mechanism or process to make such predictions. But now, this model will prove beneficial for them as they can obtain this estimate (i.e., whether the field is feasible for sugarcane production or not) about sugarcane production before contracting farmers. So far, the counting of sugarcane plants at the tillering phase was completed through a typical manual counting process, which is lengthy and requires more labor, and hence, it is an expensive process because this labor needs to be paid. The proposed model can count sugarcane and give a more accurate estimation about the yield. Counting plants during the tillering phase of sugarcane is critical for various reasons. To begin, the tillering phase is an important step in sugarcane growth since it is when secondary shoots, or tillers, sprout. Counting plants at this phase offers useful information on stand establishment and population density, which have a direct impact on crop yield potential. Furthermore, accurate plant counts allow farmers to evaluate plant health, monitor pest and disease infestations, and make informed decisions about watering, fertilization, and weed control. Farmers may optimize inputs, improve crop management procedures, and, ultimately, maximize sugarcane yields by properly counting plants throughout the tillering phase. 

The proposed work Is conducted on the tillering phase of the sugarcane using deep learning algorithms and different augmentation techniques to count and generate the dataset. Augmentation techniques have been used for the generation of the dataset, such as adding different effects like fog, rain, shadows, etc. For the detection and classification of sugarcane plants, state-of-the-art algorithms of deep learning have been utilized [5]. The transfer learning was performed on two different models, i.e., YOLOv3 [6] and Faster R-CNN [7] to compare their working and results with the proposed methodology. The proposed method uses modified architecture of Faster R-CNN for detection. The features are extracted first by using VGG-16 with Inception-v3 modules, and then the detection is performed on it. Unique IDs are assigned to the sugarcane plants, and processing has been conducted frame by frame to count the number of plants in the video.

To summarize, artificial intelligence (AI) is transforming sugarcane agriculture by enabling precision farming techniques, predictive analytics, and automated procedures. Farmers can utilize AI-powered systems to optimize resource use, monitor crop health, and more precisely estimate yields. In future, this method can enable the early identification of diseases and pests, resulting in earlier treatments and better crop management. AI-driven robots streamline harvesting procedures, lowering labor costs and increasing efficiency. Furthermore, AI assists with the following:

- AI facilitates precision farming, which optimizes resource utilization in sugarcane production.
- Predictive analytics aids in disease identification and yield prediction, which improves crop management.
- Automation using AI-powered robotics accelerates harvesting procedures, lowering human expenses.
- Sugar cane delivery is more efficient and timelier when the supply chain is optimized.
- AI increases agricultural breeding, resulting in superior sugar cane types.

## 2. Related Work

In the past few years, object detection has gained a lot of popularity [8,9]. For example, product quality checking, counting, surveillance, monitoring, etc., are some object detection applications [10,11]. The idea of counting any object through this approach is to detect the object, draw a bounding box around the object, and then count the bounded instances. Labels and valid values are provided in bounding boxes for training around objects [12]. Once the model is trained, it is tested in real-life situations.

The need for counting sugarcane seedling plants is at an all-time high according to early research [13], where researchers worked on estimating sugarcane production based on data from previous years. Data consisting of agrometeorological and spectral information were collected for the past four crop years. They used climate conditions and soil water as functions to estimate the sugarcane yield. Their sample consisted of data from 130 fields with different development stages. Their model had estimated a 69% observed yield variation in crops. 

In [14], a convolutional neural network (CNN) technique has been used to count plants in a crop. The performance of various neural networks was evaluated for plant counting with minimal modifications. The dataset used in their research was self-constructed using an unmanned aerial vehicle (UAV), which consisted of 2480 RGB images. This method was used to obtain ortho-rectified images of an entire field. The dataset was split as 80% to train, 10% for validation, and 10% for testing. The results provide a minimum Mean Absolute Percentage Error (MAPE) of 6.7% with the Inception-v3 CNN architecture. 

In another study [15], a method for plant counting using foreground extraction algorithms has been proposed. They observed the differences in spatial distributions of plants with overlapping cases. They introduced innovation to the plants with density distribution by using the density map regression. They used different approaches of deep neural networks, including patches classification, U-Net architecture, and regression task classifiers. A numerical study was also conducted to apply density map regression of palms counting and the best estimate of sugar beet based on crop canopy cover metrics. A 98.9% accuracy was obtained by doing the numerical study.

Another paper [16] overviews studies in convolutional neural networks (CNN) in plants. The main aim was to overview stress evaluation architecture, postharvest quality assessment, and plant development. In this study, the most challenging problems faced during plant phenotyping applications were demonstrated. They worked by classifying, detecting, and segmenting the images on a technical development basis. In the end, for the plant’s phenotyping purposes, they offer the different directions of CNN architecture for further research. The first one is to enhance the availability of labeled data. Some publicly available datasets were not integrated and designed for agriculture development. The second one is to develop a framework of deep learning which provides the latest deep learning (DL) techniques. It gives a typical fractionation for integration algorithms, so the new tools and models may require less effort in reinforcement learning and improving the model. Advanced DL-based data analysis can be introduced through educational programs and pieces of training for the agricultural field. The last guideline is developing and adopting CNN architecture for 3D and multimodal data processing, i.e., branch pattern, plant development understanding, and skeleton extraction.

Further, Machefer et al. in [17] have proposed a deep learning architecture combined with remote sensing for plant counting using Mask-RCNN on conventional images gathered to embody an automatic cutting edge approach. In another study [18], a procedure of object-based image analysis for UAV images to obtain information on sugarcane plant skips has been proposed. They used three different stages (1) to identify the planting rows of sugarcane, (2) to determine the surviving sugarcane within the rows of crops, and (3) to skip the removal and design of field-extent crop maps. They obtained the 0.97 coefficient of determination of the observed and estimated skip lengths; the enhanced Wilmott concordance coefficient was also calculated as 0.92, ensuring improved results. The high level of automation and adaptability was obtained by OBIA, which was beneficial for decision making, reductions in operational costs, and agricultural monitoring.

In an added study [19], a system was developed that counts the stalk width and number of the stalks. A ground robot was used, which had a camera and navigated through narrow paths. In the robot, a high-resolution stereo imager was added to capture the images for the experiments. A convolution neural network was used for detecting the object, and semantic segmentation worked with that for the stalk counting. Faster R-CNN has been implemented to calculate the width and count the stalks. A method to count the total number of plants of corn crop using a deep learning model has been presented in [20]. An aerial view of the crop was taken using multispectral sensors and unmanned aerial vehicles (UAV). From the UAV, the plants appear to be very close together and condensed. Objectifying these plants was crucial. Morphological Operators and Blob Detection were applied to overcome this problem. 

In [21], a system to automatically counts the plants using Internet of Things (IoT) devices and deep learning have been presented. Data are captured first using drones. The annotation system manages the annotation of the plants, which is an essential part of the system’s training. DL subsystem controls the inferencing and training of the system. IoT devices used in this were difficult to manage and expensive to deal with. A study to build a system that could estimate plant properties using computer vision and deep learning has been presented in [19]. They had estimated the stalk count and stalk width of Sorghum crop. For this purpose, they have extracted plant regions and calculated their density through semantic segmentation. In another latest research [22], researchers provided an improved Faster RCNN architecture with promising results for automatic detection and counting of sugarcane seedlings using aerial images. They evaluated the viability and efficiency of the proposed technology using 238 images taken from air using an unmanned aerial vehicle. 

The previous work in determining the crop yield was inspirational. However, the counting of sugarcane plants has not been conducted at the tillering phase until now, which is being proposed in this paper; the methodology is provided in the following.

## 3. Methodology

In this study, a deep learning approach is used for identifying and estimating the number of sugarcane plants. Just like humans use their brains to identify patterns and classify various types of information, deep learning algorithms can be used to accomplish these tasks. The fundamental benefit of using the deep learning techniques such as R-CNN [23], Fast R-CNN [24], Faster R-CNN [7], YOLOv3 [6], etc. is to achieve capability to perform feature engineering automatically. In the following, the newly proposed method adopted for counting sugarcane plants in the tillering phase has been discussed.

### 3.1. Proposed Solution

For the detection of sugarcane plants, modified Faster R-CNN model has been implemented as the new approach. The parameters of Faster R-CNN were fine-tuned, and the backbone architecture has been changed. The input video was passed to the model, and processing was completed frame by frame. Unique IDs were assigned to the interested objects and the features were extracted by using VGG-16 with Inception-v3 modules instead of typical VGG-16 [25], and afterwards the detection was performed. Modified Faster R-CNN was evaluated against the original Faster R-CNN and YOLOv3. 

Faster R-CNN has two modules: (1) a region proposal network (RPN) and (2) a network that detects the object based on the proposal generated by the RPN. To create the region, it uses a selective search. The image is passed to the Faster R-CNN, which generates the bounding boxes with labels. Before the region proposal, it gets the features from the backbone architecture. In [26], Liu et al. found that 80% of the time was spent on the backbone architecture so it can be improved to speed up the system. 

### 3.2. Base Network

In the conventional Faster R-CNN design, the VGG-16 network serves as the foundation for feature extraction from input images. This network handles 224 × 224-pixel pictures with three color channels (red, green, and blue). VGG-16’s convolutional layers create feature maps that capture increasingly complex visual patterns. These layers start learning low-level features like lines and edges, then graduate to higher-level properties like forms and textures. The design uses a succession of convolutional layers followed by max-pooling layers, with the max-pooling procedure reducing the spatial dimensions of the feature maps while maintaining the most relevant data. 

The mathematical formula for calculating the size of the output feature map after convolution is defined by Equation (1):

(1) $$Output\;Size=\frac{Input\;size-Filter\;size+2\times Padding}{Stride}+1$$

For max-pooling, the output size can be computed by Equation (2):

(2) $$Output\;Size=\frac{Input\;size-Pool\;size}{Stride}+1$$

**Figure 2:**
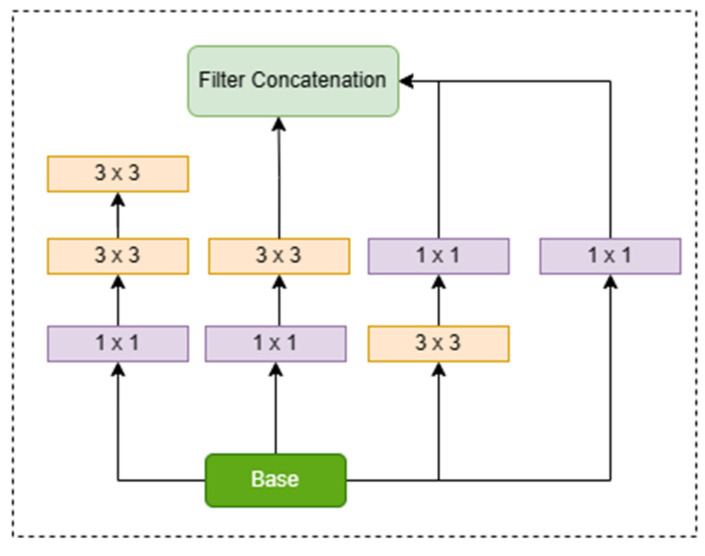
Inception-v3 Module.

In the VGG-16 network, fully connected (FC) layers follow convolutional layers for additional processing. Each of the first two FC layers has 4096 neurons, whereas the third FC layer has 1000 neurons. The hidden layers are activated using the rectified linear unit (ReLU) activation function, which introduces nonlinearity into the network. In contrast, the suggested modification integrates Inception-v3 modules into the VGG-16 architecture. Inception-v3 uses simultaneous convolutional procedures with changing kernel sizes to successfully capture scale-invariant information. This parallel processing improves the detection of tiny items in field photos, such as sugarcane, when compared to VGG-16 alone. The Inception-v3 module overcomes the constraint of VGG-16 in learning scale-variant features with a single kernel size per convolutional layer. By using kernels of different sizes in parallel, Inception-v3 improves the network’s ability to extract features at many scales, resulting in better performance in detecting tiny objects. The Inception module’s output can be expressed as a concatenation of feature maps derived from convolutional operations with various kernel sizes. The final output size would be determined by the concatenation of these feature maps from various branches, as shown in Figure 2.

As a result of overcoming the over-fitting problem, the convolution network architecture becomes wider instead of getting deeper. Large kernel sizes and small kernel sizes are used to extract global and local information, respectively. The reduced depth can be obtained by applying 1 × 1 convolution after passing it through the 3 × 3 and 5 × 5 convolution. Based on this idea, conventional Conv2D layers in GoogleNet [27] were replaced with the Inception-v3 unit. Inspired by the Inception-v3 replacement in GoogleNet [25], VGG-16 with Inception-v3 modules was used as feature extractor of modified Faster R-CNN, and each convolutional layer in VGG-16 was replaced with the Inception-v3. The two auxiliary layers in the middle of architecture have been used to overcome this problem. That is why the Inception module consists of three different loss layers, and the total loss is the sum of both losses, i.e., loss of auxiliary classifier and the actual loss.

**Figure 3:**
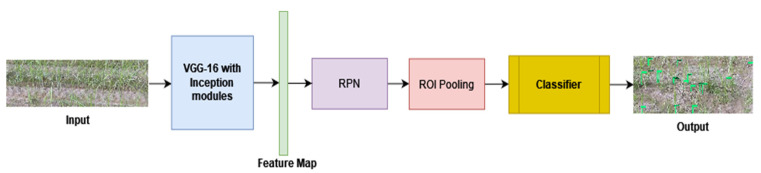
System diagram.

All types of factorizations have been applied simultaneously, and feature engineering is used afterwards in the Inception module to obtain the global and local information. There is a problem when there is an alteration in input features; the neural networks do not usually perform well. Information can be lost when feature maps are reduced, and it is known as a representational bottleneck. Smart factorization has been introduced to get rid of this problem. Two 3 × 3 convolutions are used in place of one 5 × 5 convolution, resulting in the efficiency of speed and accuracy, demonstrating that it is more efficient to use a stack of two 3 × 3 convolution with respect to computation and accuracy, as shown in Figure 2. One 3 × 3 convolution is equal to 1 × 3, and 3 × 1 convolution, which means that you must first apply a 1 × 3 convolution, and then a 3 × 1 convolution. This approach results in 1/3 lesser trainable parameters than the one 3 × 3 convolution [25]. After applying two convolutions, 1 × n and n × 1, it has been observed that when a model converges, excess training leads the model to overfitting. It has been suggested that the model will learn efficiently without having the problem of overfitting if the regularize function is utilized by the Dropout operation and Batch Normalization. Thus, an RMSProp optimizer has been used in the Inception-v3 module. 7 × 7 convolutions are factorized as three 3 × 3 convolutions, and further divide each 3 × 3 convolutional into 1 × 3 and 3 × 1 convolutions. Label smoothing has also been introduced. The primary purpose of label smoothing is to prevent the model from being overfitting. The VGG-16 with Inception-v3 modules extracts the feature map and passes it to the Region Proposal Network (RPN). Those feature maps are used as the input of the RPN network, as shown in Figure 3. A small network took the features from the previous part and gave the lower dimension features. 

The Region Proposal Network (RPN) includes two extra convolutional layers: the Regressor and the Classifier. We represented the input feature mappings to these layers as F, which have dimensions H×W×C, where H is the height, W is the width, and C is the channel count.

In the Classification layer, the Classifier predicts whether the suggested region belongs to a class or the background. Let P_i_ denote the expected chance that anchor i belongs to the object class.

This can be expressed as Equation (3):

(3) P_i_ = sigmoid (W_c_ × F_i_ + b_c_)

where W_c_ is the weight matrix and b_c_ is the bias vector for the classifier layer.

The Regressor layer determines the bounding box coordinates for each anchor. Δt_i_ represents the projected adjustments to anchor i coordinates for bounding box regression. It can be represented by Equation (4):

(4) Δt_i_ = W_r_ × F_i_ + b_r_

where W_r_ is the weight matrix and b_r_ is the bias vector for the regressor layer. 

Both layers use nine anchors, resulting in nine predictions for each position in the feature map. Proposals are then passed through the RoI pooling layer, which uses max-pooling with a 7 × 7 window size and 512 channels to generate fixed-size feature maps. The RoI pooling layer’s outputs, together with the Inception-v3 feature maps, receive input via fully linked layers. Because there is just one class (sugarcane), the suggested model employs a sigmoid function to predict class scores. Theoretically the class prediction is represented by Equation (5):

(5) Probability of sugarcane = sigmoid (W_s_ × RoI features + b_s_)

In bounding box regression, the SVM classifier predicts the class while the regression layer modifies the bounding box around the item. The number of bounding boxes in photos is counted, whereas in videos, each item is granted a unique ID for tracking reasons, which is subsequently counted.

## 4. Experiments and Results

### 4.1. Materials and Methods

Sugarcane agriculture requires precise weather and soil types for maximum growth. It flourishes in tropical and subtropical regions with temperatures ranging from 20 °C to 30 °C throughout the growing season, requiring well-distributed rainfall of 1000 to 1500 mm per year or supplementary irrigation. Adequate sunshine is required for photosynthesis and sugar buildup. The crop prefers well-drained, fertile soils, such as sandy loam or loamy soils, that hold moisture without becoming saturated. For best nutrient availability, soil pH should be between 6.5 and 7.5. Overall, sustaining these conditions is critical for effective sugarcane farming, as it ensures healthy plant growth, high yields, and desirable sugar content in the harvested crop. South Asian regions jointly contribute to sugarcane production, with different climatic conditions, soil types, and water availability influencing growing practices and productivity levels. Pakistan is located in South Asia, and sugarcane agriculture is an important part of the country’s economy, contributing to agricultural output and providing job opportunities for millions of people. The crop requires a warm climate with evenly distributed rainfall or access to irrigation.

Data for the current study were collected using a camera mounted on a quadcopter in a sugarcane field of Punjab, Pakistan. The cultivation of the sugarcane field was performed in the fall season, and the videos were collected after two months when the sugarcane plant was in tillering phase. At that stage, sugarcane was not fully grown, but the plant’s cane had come out from the ground which was needed for this work. Our newly developed dataset is publicly available [28]. 

This research work focuses on the counting of the sugarcane plant at the tillering phase. For dataset training and testing, the split of the data is 70–30, respectively. For evaluation metrices, Equations (6)–(9) represent accuracy, precision, recall, and *F*1 score, respectively. In equations *TP* represents True Positive, *TN* represents True Negative, *FP* represents False Positive, and *FN* represents False Negative.

(6) $$Accuracy=\frac{TP+TN}{TP+FP+FN+TN}$$

(7) $$Precision=\frac{TP}{TP+FP}$$

(8) $$Recall=\frac{TP}{TP+FN}$$

(9) $$F1Score=\frac{2\times (Recall\times Precision)}{\left(Recall+Precision\right)}$$

### 4.2. Data Preparation

A total number of 373 number of frames were taken out for the labeling after the preprocessing. A labeling tool, “LabelImg”, was used to label the images. The cane part below the leaves was labeled. Annotating the images was an arduous task as the cane part was very small. For labeling, it requires precision and accuracy for drawing a bounded box around the cane. As for 373 images of data, approximately 18,650 bounding boxes were drawn [29]. A total of 3730 images were generated using effects mentioned earlier. The bounding boxes were assigned class name “sugarcane”. The cane part of the sugarcane was brown greenish-yellow, annotated for the training of the model. Preprocessing of the data was carried out to filter out noise while keeping useful features of the field. From the videos, frames were extracted at a rate of 20 fps frame per second. These frames were cleaned by removing the blur frames during frame extraction method. A single frame had 40–60 sugarcane sticks. Since the plants near to the camera were clearer and more visible, images were cropped to remove plants away from camera.

The dataset was generated from the sugarcane field. The dataset was not diverse and sufficient as the deep learning problem requires a large amount of data, so the dataset was augmented using different effects. Effects like fog, rain, sun, and shadows were also added to the dataset. In order to make data more challenging and realistic, they were augmented using blur, motion blur, horizontal flip and noise effects to increase the number of images. These effects were applied as a single unit and also in combination with each other. Using imgaug library [30] provides fine-grained control over augmentation settings and may be easily integrated into data pipelines. Imgaug provides a variety of tools for simulating environmental conditions and lighting effects in photos. For fog, use Gaussian blur to blur the image, Multiply to lower the contrast, and Contrast Normalization to modify brightness. Rain effects are created with Dropout and CoarseDropout to remove pixels and Motion Blur to add streaks. For lighting modification, use Multiply for brightness control, Additive Gaussian Noise for random noise, and Add for overlaying light sources. These methods provide a flexible toolkit for creating various atmospheric effects and enriching image collections for machine vision tasks.

### 4.3. Results and Discussion

**Figure 4:**
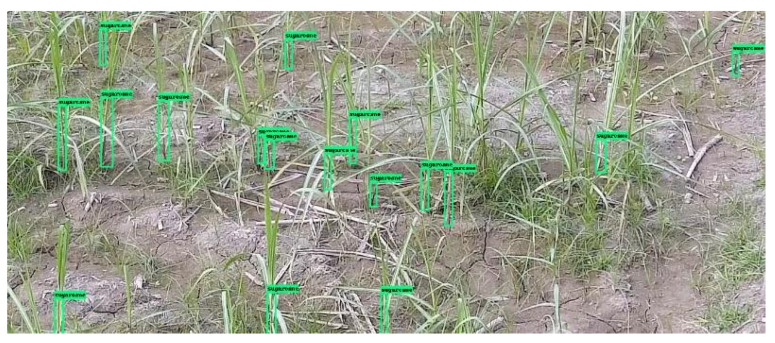
Detection results on the sample image by modified Faster R-CNN.

The dataset was trained on different models to compare their performance and results. Experimentation was performed on the YOLOv3 and Faster R-CNN using transfer learning method as well as on the newly modified architecture of Faster R-CNN on the generated dataset of the sugarcane plants in this study. The result of the sugarcane detection using the proposed methodology has been shown in Figure 4.

**Figure 5:**
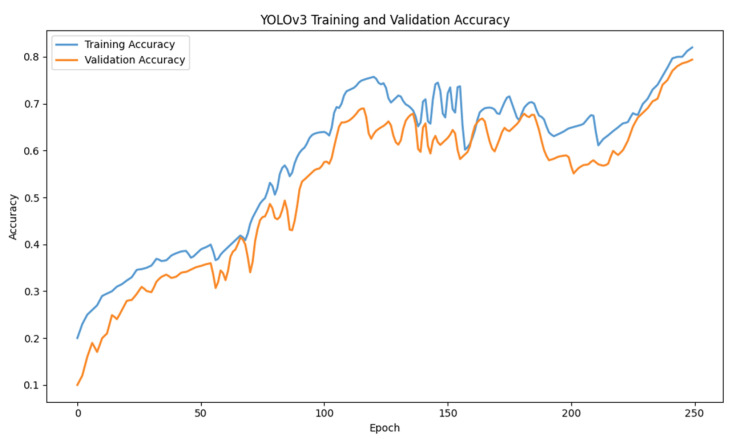
YOLOv3 accuracy graph.

**Figure 6:**
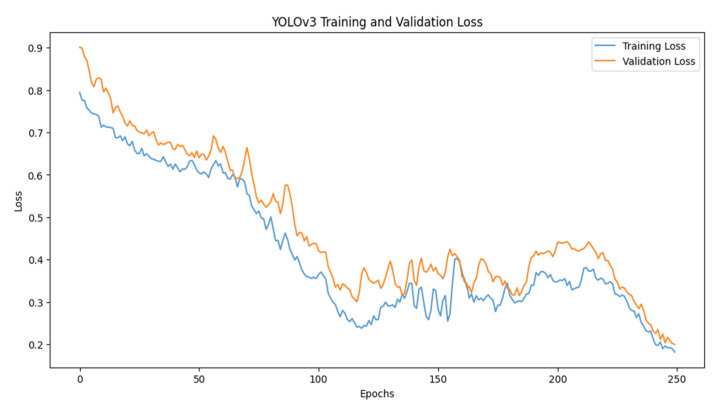
YOLOv3 error loss graph.

For training YOLOv3, the epochs were kept at 25 with a batch size of 16. The loss started to decrease as the epochs increased. The accuracy of the model also tends to increase as the number of epochs increases. The x-axis of the graph shows the number of epochs, and y-axis shows the accuracy. The accuracy achieved while training YOLOv3 was 78.13%. Figure 5 and Figure 6 depict accuracy and loss graphs, respectively. 

**Figure 7:**
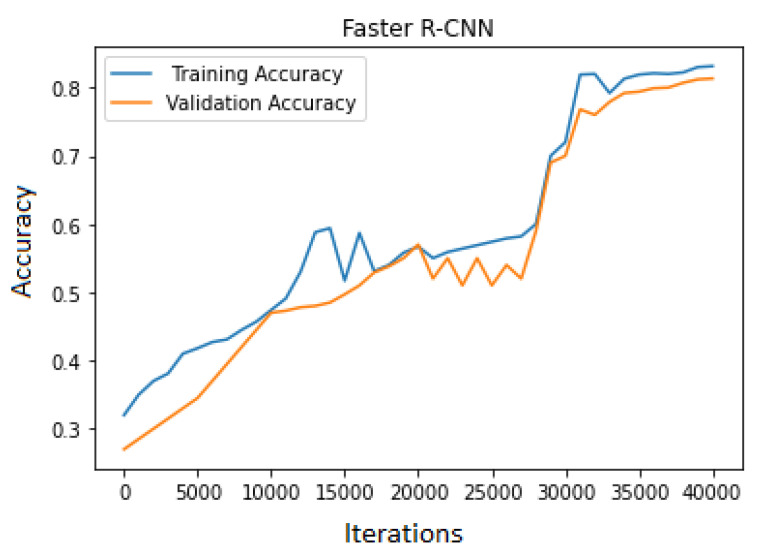
Faster R-CNN accuracy graph.

**Figure 8:**
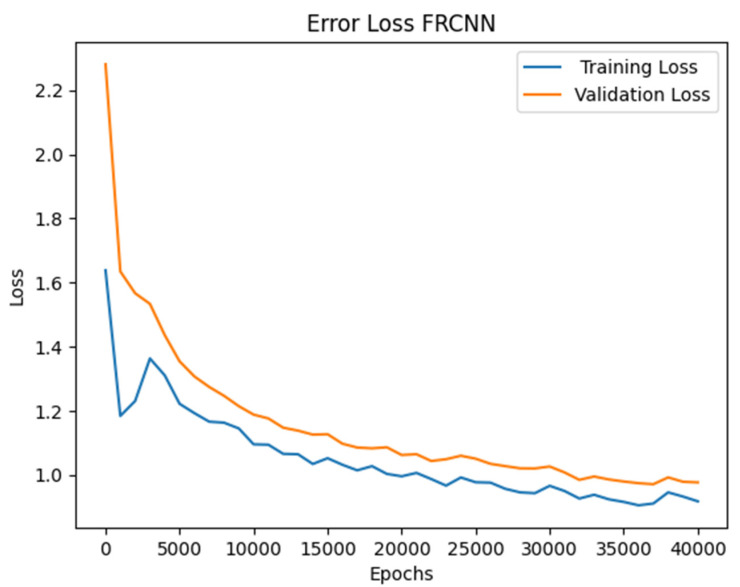
Faster R-CNN error loss graph.

For training Faster R-CNN, transfer learning with 40,000 iteration and a batch size of 16 was used to train the model on the dataset. The training and validation accuracy trends were increasing but at a slow learning rate. The training accuracy was high compared to the validation. As the number of epochs increased, the loss started to decrease from 11.02 to 0.31. This model’s learning rate was slow compared to YOLOv3. The accuracy achieved on the dataset was 81.33%, as shown in Figure 7, and the loss graph is represented in Figure 8.

**Figure 9:**
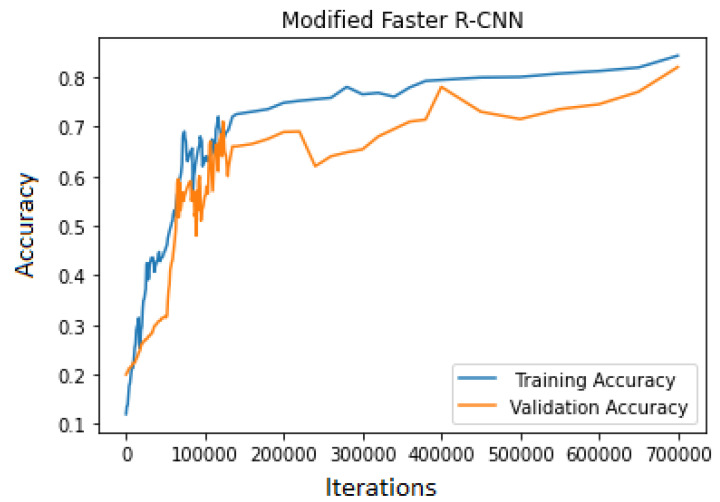
Modified Faster R-CNN accuracy graph.

For the proposed modified Faster R-CNN training, the model needed to be trained from scratch. The model was trained from scratch and 70,000 iterations were required to be run. The model was implemented in python language with different libraries, i.e., Tensor flow, NumPy, Sci-kit learns, etc.; the model was trained on the core I7 with 32 GB of RAM having Nvidia 1080 Ti GPU. The accuracy of validation and training data was checked multiple times by stopping the training process in order to avoid overfitting; the maximum training and validation points are shown in Figure 9. 

**Figure 10:**
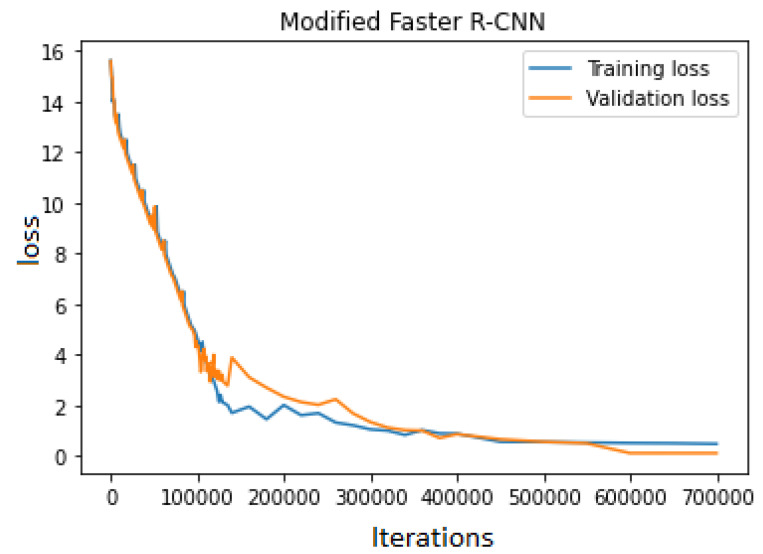
Modified Faster R-CNN error loss graph.

The training continued until the error loss decreased. The proposed model was trained on 3730 images with the label name sugarcane. The proposed model achieved an accuracy of 84.3% for training data and 82.10% for validation data. While training the model, the error rate was relatively high. The error loss decreased rapidly, but after some time, the loss was stochastic, as shown in Figure 10.

The methodology proposed showed better results on the dataset. The validation and training loss were also less compared to the YOLOv3 and Faster R-CNN. The comparison of the accuracy is shown in Table 1. The accuracy of the proposed methodology was high compared to the other models.

The YOLOv3 took the least time to train compared to the Faster R-CNN and the proposed model. The proposed model also took more time as compared to others but achieved better accuracy than others. In object detection, Intersection over union (IoU) is the most feasible approach for the evaluation. Using a degree of overlapping area, we calculated the difference between ground truth and the bounded box generated by the model. For the assessment of the model, we have used the F1 score, precision, and recall. We validated the system by using 200 images, and almost every image had 45–50 sugarcane plants. The confusion matrix evaluates the system as it enumerates the true positive and false positive results of the system. The proposed model gave the 6340 sugarcanes a true positive result. The F1 score, recall, and precision of sugarcane class on different models are shown in Table 2.

**Figure 11:**
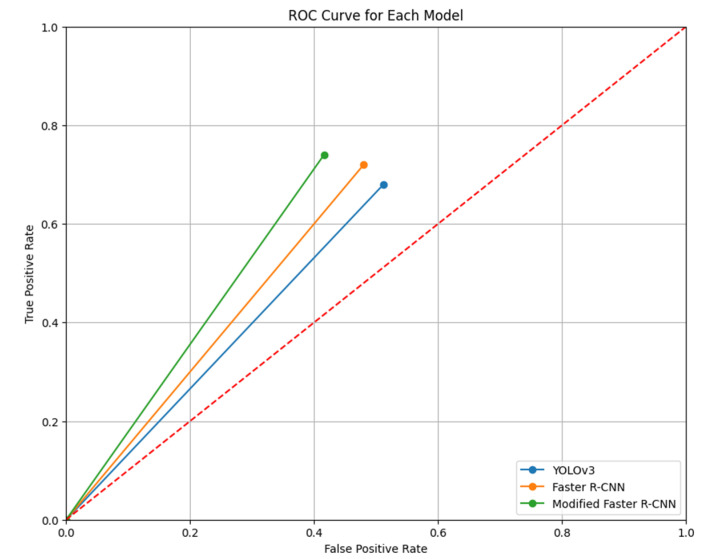
ROC curve for each model.

Another method of evaluation that was performed was the receiver operating characteristic (ROC) curve. Figure 11 depicts the results, which show that the simplified ROC curve visualization above contrasts the performance of three object detection models—the YOLOv3, Faster R-CNN, and Modified Faster R-CNN—using a unique point to represent each model’s performance based on a hypothetical single threshold. The models move from (0,0), where no instances are classified as positive, to their specific performance coordinates: YOLOv3 ends at (FPR: 0.512, TPR: 0.680), Faster R-CNN at (FPR: 0.480, TPR: 0.720), and Modified Faster R-CNN at (FPR: 0.416, TPR: 0.740). The closer a point is to the top left corner, the better, indicating higher sensitivity (TPR) and specificity (1-FPR). This plot demonstrates that, under these conditions, the Modified Faster R-CNN outperforms the other models with the lowest FPR and highest TPR, indicating its superior ability to correctly identify positive instances while minimizing false positives.

## 5. Conclusions and Future Work

This research focuses on the counting of the sugarcane plant at the tillering phase. The dataset was generated from the sugarcane field. The dataset was not diverse and sufficient, as the deep learning problem requires a large amount of data, so the dataset was augmented using different effects. These effects include motion blurring, adding shadows at various points in the images, and flipping the images. The effects of weather were also added, like fog, sun, and rain, to make the dataset more diverse. The dataset was trained on different models, like the YOLOv3 and Faster R-CNN, using transfer learning, to compare them with the proposed modified model of Faster R-CNN. For the proposed model, the architecture of Faster R-CNN was revised. Features were extracted using VGG-16 with Inception-v3 modules instead of using VGG-16. For counting the number of plants in the video, unique IDs were assigned to the distinguished objects, and processing was carried out frame by frame. The proposed methodology achieved an accuracy of 82.10%. The YOLOv3 model was faster in terms of time than others, but the proposed model outperforms the rest of the models from an accuracy perspective. We have opened up a new research area by estimating sugarcane plant counts at the tillering phase. These findings highlight the potential for AI to transform sugar cane production by providing precise monitoring, data-driven insights, and superior crop management tactics. Furthermore, the study opens up new avenues for future research, such as refining AI algorithms for even greater accuracy and investigating segmentation techniques to reduce background interference in image processing, all of which contribute to sustainable and efficient agricultural practices in the sugar cane industry. Next steps in the use of AI in sugarcane cultivation include a variety of new approaches; these include investigating sophisticated data augmentation techniques to generate more diversified datasets, integrating remote sensing data for larger-scale monitoring, and developing real-time decision support systems to enable prompt actions. Furthermore, climate change adaptation requires predictive modeling and adaptive crop management approaches. Collaborative knowledge-sharing platforms can boost innovation, while field operations beyond plant counting can be automated to reduce processes even further. Exploring explainable AI strategies adapted to agriculture promotes transparency and trust in AI-driven suggestions. By following these future approaches, researchers might improve productivity, sustainability, and resilience in sugar cane agriculture, benefiting farmers and the agricultural sector as a whole.

## Figures and Tables

**Table 1:** Accuracy of Different models.

Methods	Dataset	Accuracy
YOLOv3	Sugarcane dataset	78.13%
Faster R-CNN	Sugarcane dataset	81.33%
Modified Faster R-CNN	Sugarcane dataset	82.10%

**Table 2:** Evaluation of different models.

Methods	Precision	Recall	F1-Score
YOLOv3	0.57	0.68	0.62
Faster R-CNN	0.60	0.72	0.65
Modified Faster R-CNN	0.64	0.74	0.68

## Data Availability

Dataset is available at the following: Ubaid, Talha; Javaid, Sameena (2024), “Sugarcane Plant in Tillering Phase”, Mendeley Data, V1, https://doi.org/10.17632/m5zxyznvgz.1. Ubaid, Talha; Javaid, Sameena (2024), “Annotated Sugarcane Plants”, Mendeley Data, V1, https://doi.org/10.17632/ydr8vgg64w.1.
